# Proteomics of prostate cancer serum and plasma using low and high throughput approaches

**DOI:** 10.1186/s12014-024-09461-0

**Published:** 2024-03-12

**Authors:** Ghaith M. Hamza, Rekha Raghunathan, Stephanie Ashenden, Bairu Zhang, Eric Miele, Andrew F. Jarnuczak

**Affiliations:** 1 Discovery Sciences, R&D, AstraZeneca, Cambridge, UK; 2 Bioanalytical and Biomarker, Prevail Therapeutics, Wholly Owned Subsidiary of Eli Lilly and Company, New York, NY 10016 USA

**Keywords:** Biomarkers, Diagnostic, Mass spectrometry, Plasma, Serum, Proteomics, Biofluids, Prostate cancer, TMT, FAIMS, BOXCAR

## Abstract

Despite progress, MS-based proteomics in biofluids, especially blood, faces challenges such as dynamic range and throughput limitations in biomarker and disease studies. In this work, we used cutting-edge proteomics technologies to construct label-based and label-free workflows, capable of quantifying approximately 2,000 proteins in biofluids. With 70µL of blood and a single depletion strategy, we conducted an analysis of a homogenous cohort (*n* = 32), comparing medium-grade prostate cancer patients (Gleason score: 7(3 + 4); TNM stage: T2cN0M0, stage IIB) to healthy donors. The results revealed dozens of differentially expressed proteins in both plasma and serum. We identified the upregulation of Prostate Specific Antigen (PSA), a well-known biomarker for prostate cancer, in the serum of cancer cohort. Further bioinformatics analysis highlighted noteworthy proteins which appear to be differentially secreted into the bloodstream, making them good candidates for further exploration.

**Supplementary Information:**

The online version contains supplementary material available at 10.1186/s12014-024-09461-0.

## Introduction

Early advancements in the field of proteomics have sparked an interest in its application towards analysis of blood as an easily accessible biofluid for clinical applications. Biomarker discovery, validation and diagnostics studies have successfully been demonstrated using the technology [1–4]. However, blood is highly complex and contains the cellular components of erythrocytes, thrombocytes, and lymphocytes and liquid component of plasma. Serum is the liquid portion of the blood after clotting is initiated and cellular components are removed. Complete quantification of the proteome across the blood’s full dynamic range has been challenging. Blood and subsequently plasma spans a minimum of 10 orders of magnitude and, historically, most mass spectrometry-based approaches allowed an estimated 5 orders of magnitude in coverage [1]. As the samples are complex, they need to be extensively processed. This is commonly done using immunoaffinity depletion of highly abundant proteins, such as serum albumin, followed by chromatographic peptide prefractionation [2, 5]. In addition to the laborious sample preparation, instrument data acquisition can be relatively slow making it more difficult to apply discovery proteomics in a truly high-throughput manner necessary to generate large cohort datasets. However, recent advancements with the Orbitrap Eclipse technology have enabled higher sensitivity, speed, and robustness to extend the coverage and increase the detection limits. Notably, improvements in performance of mass analysers with increased sequencing speed and optimal ion movement and usage alongside specialized instrumentation designed to eliminate contaminations and interferences, such as high-field asymmetric waveform mobility spectrometry (FAIMS Pro), have been developed [6, 7]. Novel MS acquisition strategies have also taken spotlight, such as Data Independent Acquisition (DIA) [8, 9] and BoxCar acquisition [10]. In addition to orbitrap-based technology other advanced platforms are available. Notably, TIMS-TOF (Trapped Ion Mobility Spectrometry - Time-of-Flight) technology, with the Parallel Accumulation-Serial Fragmentation (PASEF®) method [11, 12] and Scanning SWATH (Sequential Window Acquisition of All Theoretical Fragment Ion Mass Spectra), with the Zeno trap [13, 14]. Those, together with optimized one pot sample preparation workflows, designed to eliminate sample loss, allow significantly increased throughput [15].

Prostate cancer (PCa) is one of the most frequent cancer diagnoses for men and detection may require active investigation [16]. It is a genomically and phenotypically heterogeneous disease displaying different clinical behaviour. Several risk factors for PCa have been identified [17] and Prostate Specific Antigen (PSA) screening allows diagnosis of the disease. Other tools for screening, as well as diagnosis and surveillance include digital rectal examination and multiparametric Magnetic Resonance Imaging [18]. However, alternative diagnostic tests and biomarkers are required due to low specificity of PSA. PSA positive predictive value is below 50% and reports suggest it is of limited use in early PCa detection [19].

Multiple discovery mode proteomics studies have explored potential biomarkers of PCa, including in serum-based assays [20–22]. The existing studies have made significant progress in identifying potential PCa biomarkers. However, they faced limitations related to validation, heterogeneity, technical constraints. New proteomics studies could address these shortcomings by exploring additional markers, alternative proteomic techniques, and conducting rigorous validation across a broader and more diverse patient population. For example, several suggested markers, including SGCd, SRC, CST3, and VWA5B2, did not show significant differences in abundance across the disease groups when validated with ELISA [22]. This limited validation success suggests that a broader range of markers may be required to improve diagnostic accuracy and sensitivity. In addition, PCa is known for its clinical and molecular heterogeneity. This might contribute to the difficulty in identifying commonalities with pooled samples. Therefore, additional proteomics studies are needed to either capture the full spectrum of PCa heterogeneity or, as in our case, focusing on a homogenous and well characterised cohort.

In this work we contribute to previous efforts and show that dozens of proteins are differentially expressed between blood of PCa patients with medium-grade disease and control (healthy) individuals. We achieved this with analytical workflows that can comprehensively and reproducibly quantify proteins from blood specimens.

## Results

### Development and optimization of proteomics workflows for blood-based samples

**Fig. 1:**
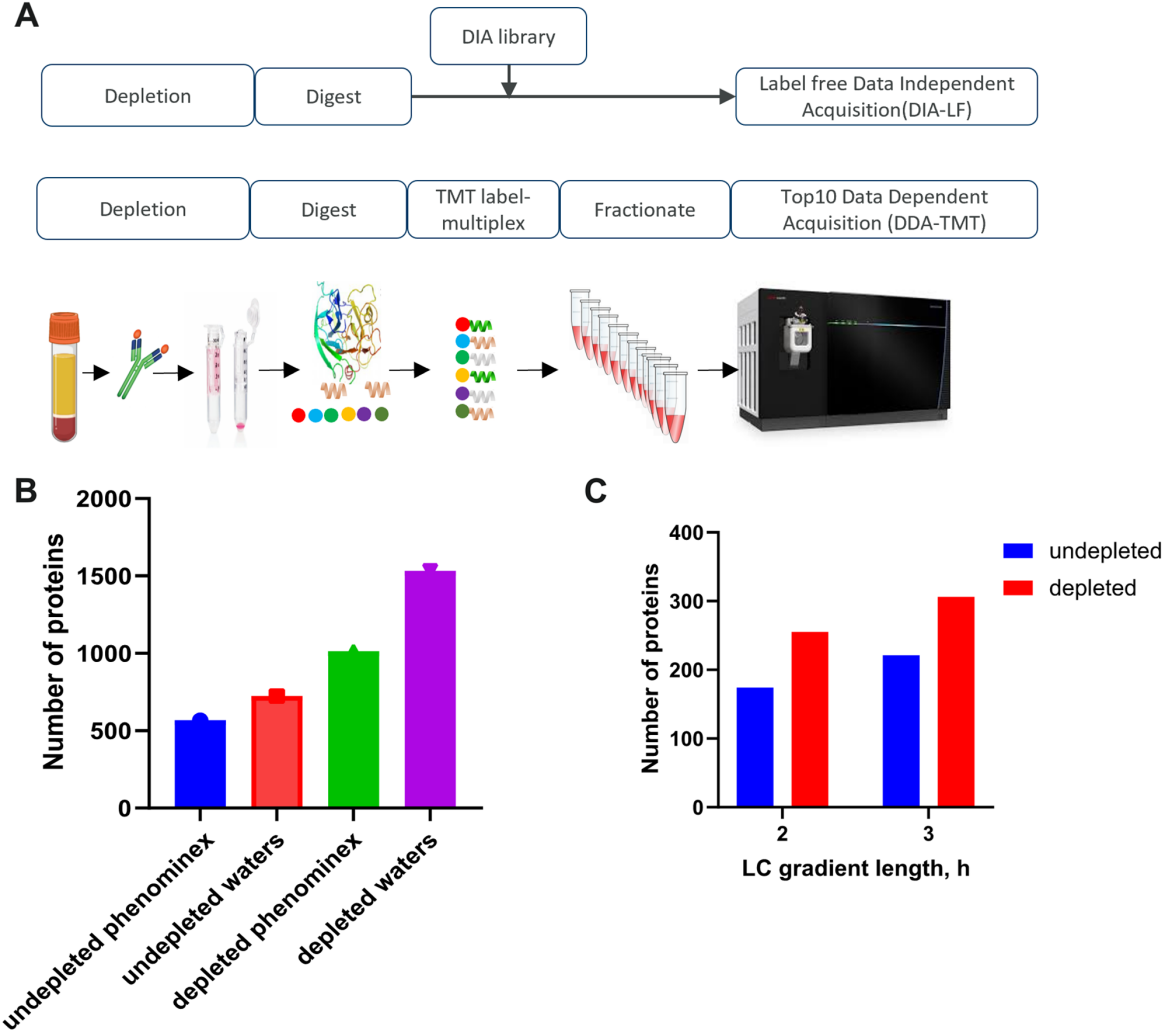
Optimized workflow for global deep profiling of plasma and serum (**A**) Overview of blood-based proteomics workflows for biomarker discovery (**B**) and (**C**) Key method development metrics for the DDA-TMT workflow

We established and characterized two proteomics workflows to quantify disease-relevant blood proteins. For this purpose, we used commercially acquired serum and plasma samples from subjects with PCa and healthy individuals. We chose to generate and compare proteomics data in two modes: low-throughput labelled (DDA-TMT) and higher-throughput label free (DIA-LF) (Fig. 1A). The low-throughput workflow involved sample labelling with tandem mass tags, offline fractionation and differential ion mobility (FAIMS) with real-time MS3 search on the Orbitrap Eclipse. The higher throughput workflow employed label free sample preparation with no fractionation and data independent BoxCar acquisition strategy on the Orbitrap Exploris 480. We applied the approach to discover proteins that are differentially expressed in subjects with PCa and could therefore represent blood-based disease biomarkers.

To process the biofluids, we tested PreOmics and EasyPep sample preparation kits in workflows including abundant protein depletion (antibody based top14 depletion) or without the depletion step. The depletion step reduced sample complexity and increased protein identification rates by $\sim$ 80%. The two commercial kits tested showed similar performance as assessed by number of identified peptides and digestion efficiency (between 5-8% missed cleavage rate). We further tested performance of peptide separation using Waters versus Phenomenex columns of comparable length but varied particle size. We observed 28% increase in the number of proteins identified in undepleted plasma and a 51% increase in the number of proteins identified in depleted plasma using optimized gradient with the Waters column (Fig. 1B). Additionally, increasing the LC-gradient length, from 120 to 180 min, resulted in $\sim$ 20% improvement in number of protein identifications (Fig. 1C).

Ultimately our best optimised method started with 70uL of biofluid that allowed extraction of 100 ug of protein. The DDA-TMT workflow allowed deep proteome coverage, albeit with a lower throughput. We estimate 16 samples could be processed per day with 60 h of acquisition time. The DIA-LF was significantly higher throughput (16 samples prep per day, 2 h acquisition per sample) but yielded roughly 50% lower coverage.

### Application of differential ion mobility (FAIMS) improves PSM identification rates

**Fig. 2:**
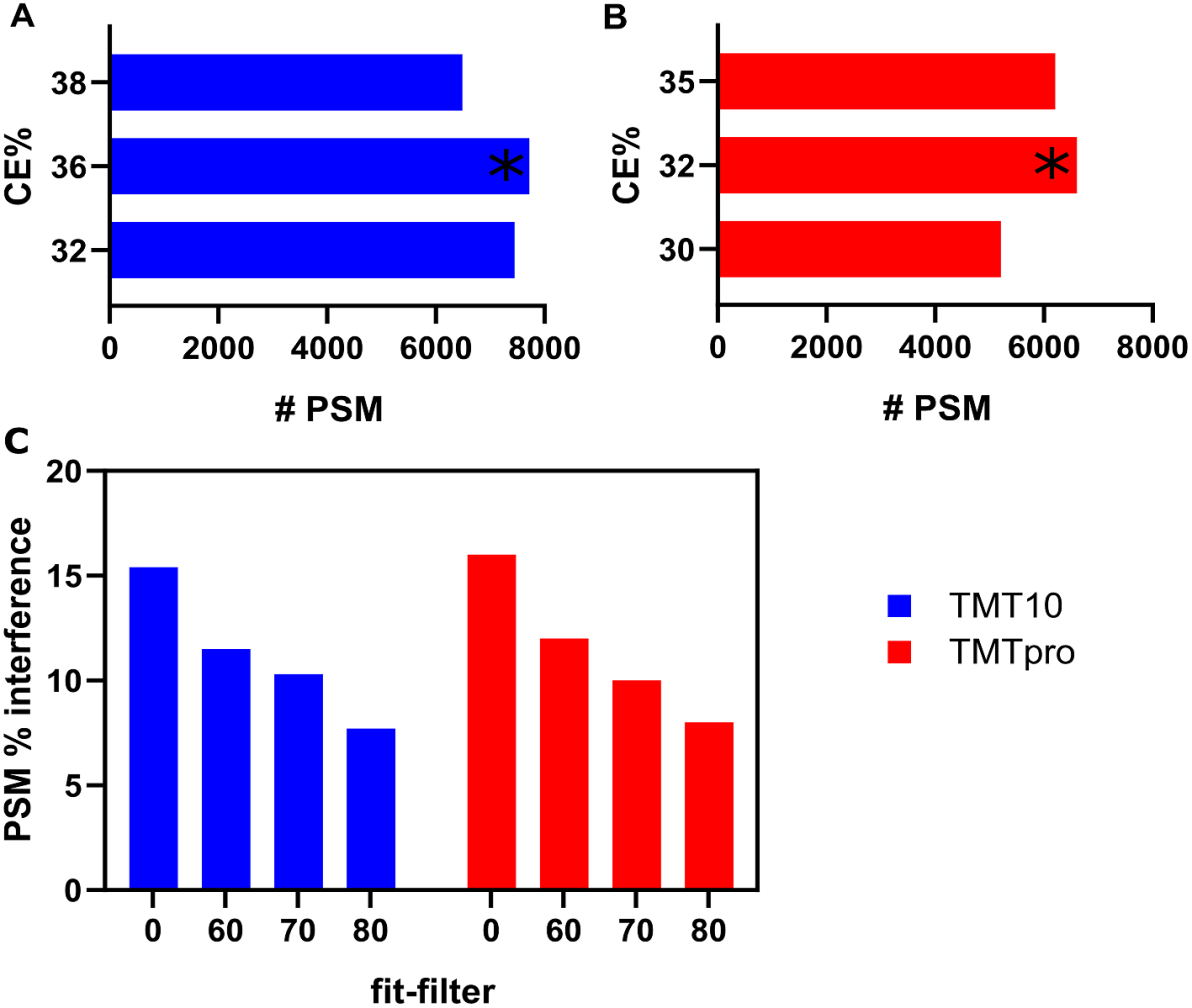
FAIMS parameters optimization. (**A**) and (**B**) effect of varying the collision energy (CE) on Peptide-Spectrum Matches (PSM). CE is adjusted to control the extent of fragmentation or dissociation of the ions. By increasing or decreasing the collision energy as a percentage of the maximum, one can influence the type and extent of fragmentation that occurs. (**C**) effect of varying the fit filter settings on PSM identification rates. Fit filter is a method used to select precursor ions with a defined precursor specificity. It does so by comparing the observed isotopic envelope (the distribution of isotopes for a given ion) to a theoretical isotopic envelope

We optimized the collision energies and fit-filter to select the optimal parameters for peptide fragmentation of TMT10 and TMTpro 16 Plexes as well as fit-filter for least Peptide-Spectrum Matche (PSM) interference for the Data Dependent Acquisition (DDA-TMT) workflow (Fig. 2). Fit filter is a method used to select precursor ions with a defined precursor specificity. It does so by comparing the observed isotopic envelope (the distribution of isotopes for a given ion) to a theoretical isotopic envelope. The normalized similarity between these envelopes must meet a user-defined fit threshold to trigger a new MS2 scan. In this experiment, four different thresholds were tested. As shown in Fig. 2A and B, we observed that collision energy (CE) of 36% was ideal with maximum PSMs for TMT10 labeled plasma while CE of 32% was ideal with maximum PSMs for TMTpro 16 Plex labeled plasma. At CE of 36%, we varied the fit-filter values as shown in Fig. 2C and observed a drop in PSM % interference from 15 to 8% for TMT10 at a fit-filter value of 80 and at CE 32% and fit-filter 80 observed a drop in % PSM interference from 16 to 8%, which was used as the optimal value in the downstream experiments.

### Characterization of plasma and serum proteome

**Fig. 3:**
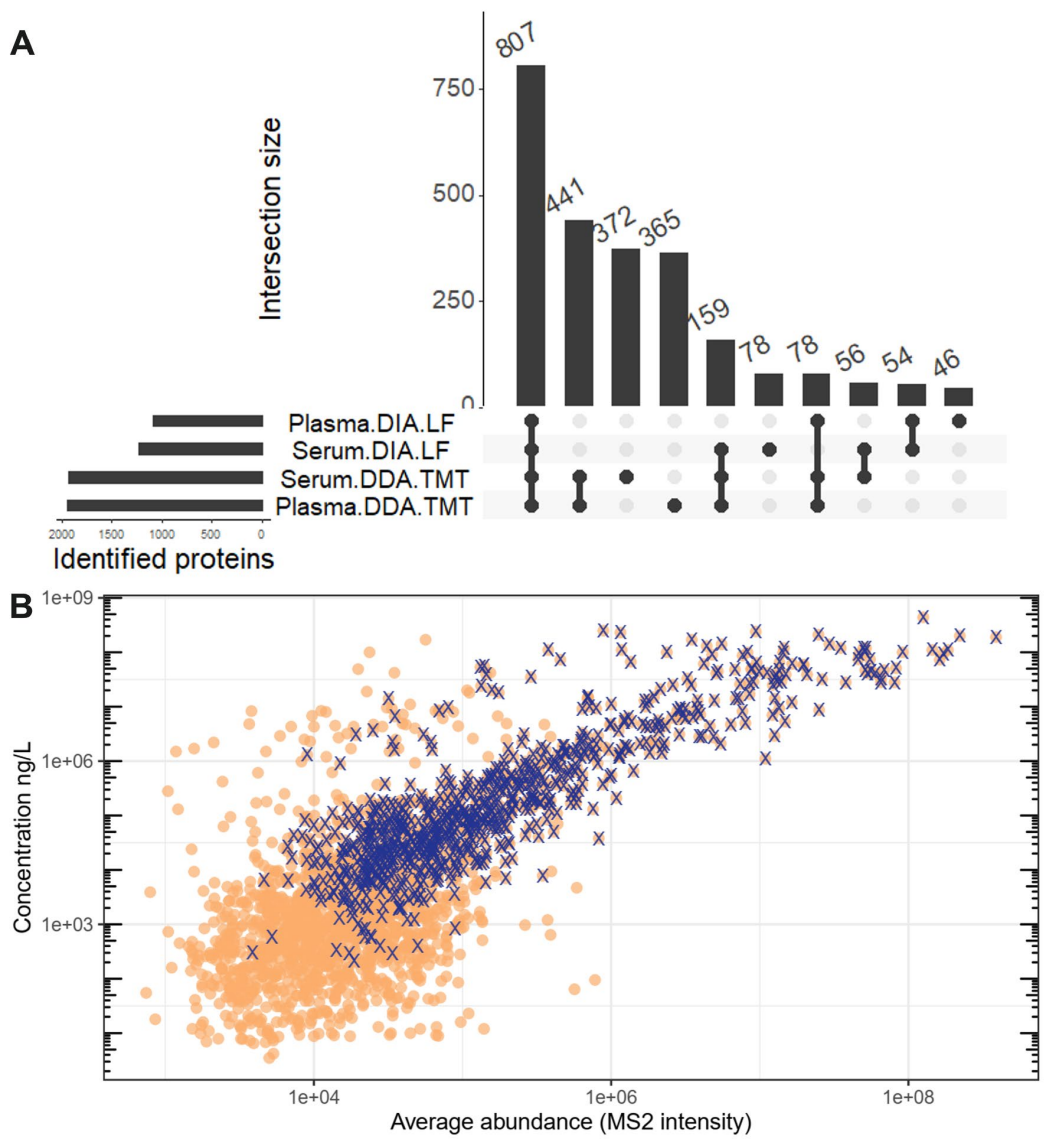
(**A**) Upset plot showing overlap of identified proteins in serum and plasma samples in both DIA-LF and DDA-TMT mode. 807 proteins were overlapping between all samples, while 441 were detected in only DDA-TMT workflow. (**B**) Plot showing the dynamic range of the detectable proteome. Average protein intensity (x-axis) versus estimated concentration in blood according to Human Protein Atlas [23]. Core proteome identified in all the samples is highlighted with “X”

Using our optimised workflows, we acquired data from 16 serum and 16 plasma PCa patient samples and matching number of healthy volunteers (32 samples total). Due to limitations of TMT tagging this portion of the study included only 15 biosamples. In total, 2939 proteins were identified at least once across all samples (Fig. 3A). On average, 817 and 987 proteins were detected per sample in plasma and serum with DIA-LF. Using DDA-TMT 1589 and 1527 were detected in plasma and serum respectively. A core proteome quantified across all samples consisted of 807 proteins. (Fig. 3A). We assessed the dynamic range of the quantified proteome by plotting the average MS intensity versus the estimated blood concentrations reported in Human Protein Atlas [23] (Fig. 3B). As expected, the measured MS intensity did not correlate well with absolute abundance, particularly for the lowest abundant proteins. The DDA-TMT workflow was able to quantify proteins with abundances below 10 ng/L. In this range some of the lowest abundance proteins were Utrophin estimated at 4.2 ng/L or Interleukin 16 at 7.3 ng/L. The core proteome, which was reproducibly detected across both workflows, however, had a much lower dynamic range and required the protein to have a minimum concentration in the 100s ng/L range. For example, EIF4B at 340 ng/L or GRB2 at 400 ng/L were the lowest detected.

### Differential expression analysis between healthy and PCa samples

We performed pairwise comparisons between healthy and PCa disease samples using limma package [24]. To control for confounders and ensure the protein expression changes we determine are specifically associated with PCa rather than age or weight-related factors, we included age and Body Mass Index (BMI) as covariates in the limma regression model. All other clinical features were closely matched between the PCa and healthy samples (see Supplementary File 1 containing all sample metadata). False discovery rate (FDR) was controlled using Benjamini-Hochberg correction and we required differentially expressed proteins (DEPs) to have an FDR p.value < 0.05. The full list of proteins and their differential expression values are available in Supplementary File 2 (Supplementary_File_2_all.data.xlsx).

**Fig. 4:**
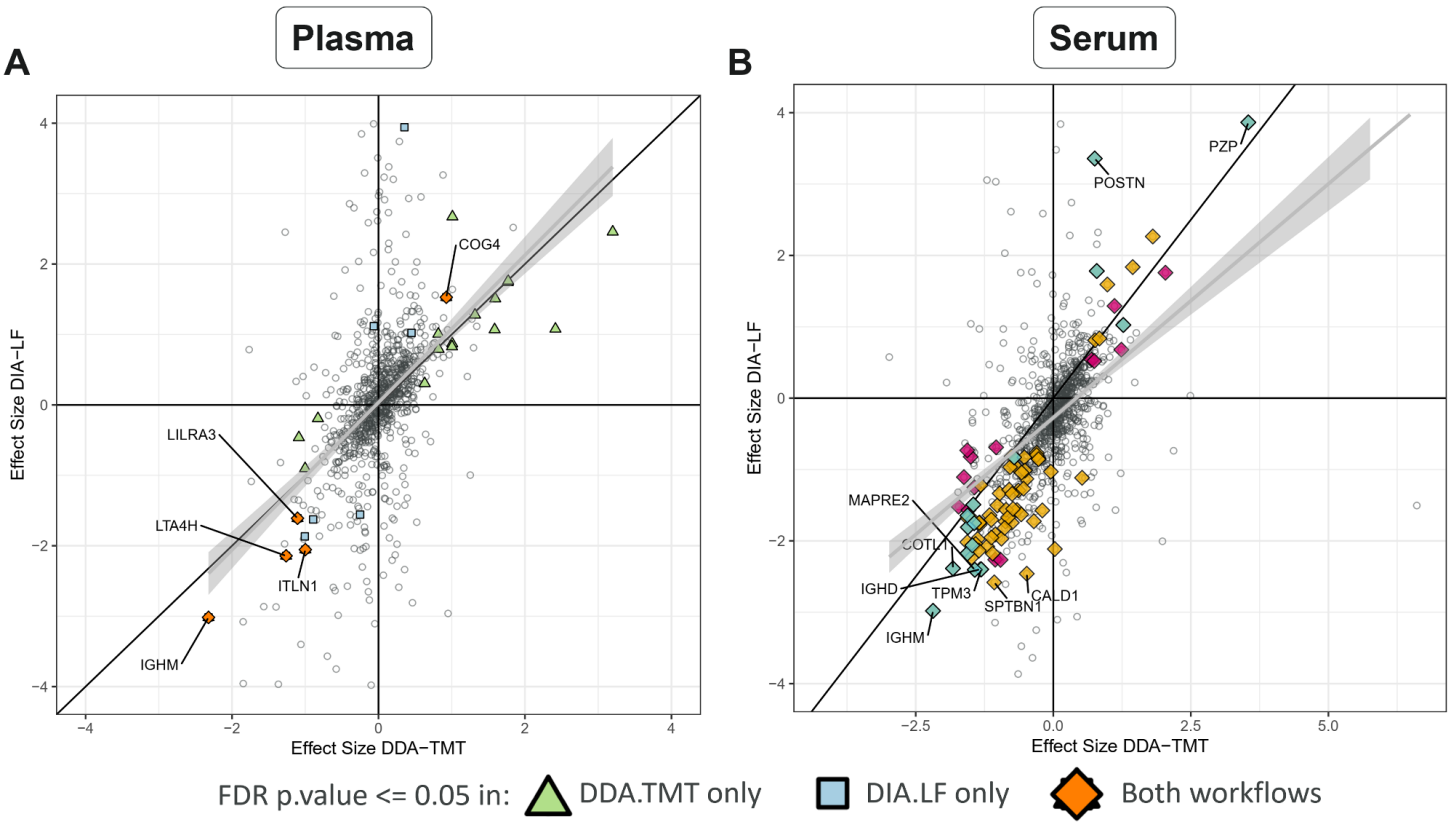
Correlation of the calculated PCa vs. healthy blood expression changes between DDA-TMT and DIA-LF workflows in (**A**) plasma and (**B**) serum. Triangles correspond to DEPs (FDR-adjusted *p*-values < 0.05) identified in only DDA-TMT workflow, squares to DEPs identified in only DIA-LF workflow and diamonds to DEPs identified by both workflows. Effect size is the log2 fold-change (FC) of PCa/healthy. Names of the most confidently called proteins by both workflows are highlighted

In plasma DDA-TMT, 14 proteins were down-regulated and 20 up-regulated, while in plasma DIA-LF, 10 were down and 4 up-regulated. For the 5 DEPs called by both workflows, we saw a very good correlation between their fold changes (*R* = 0.97, p-value = 0.004) (Fig. 4A).

LTA4H (P09960) was downregulated in PCa patient’s plasma with the greatest statistical confidence using both workflows and with at least 2-fold change. Other proteins apparently downregulated in PCa plasma were IGHM, ITLN1 and LILRA3. Among the up-regulated proteins, COG4 was identified by both workflows.

Examining serum results, a much greater numbers of differentially expressed proteins, compared to plasma, was observed. Serum DDA-TMT workflow identified 105 down-regulated and 56 up-regulated proteins. While DIA-LF results showed 141 and 38 DEPs that were down and up-regulated, respectively. From gene set enrichment analysis, the most statistically confident gene ontology terms suggested downregulation of cytoskeletal protein binding. Comparing the effect size determined in serum DDA and DIA experiments, showed a correlation of *R* = 0.93, p-value < 2.2e-16 for the 58 DEPs overlapping in both workflows (Fig. 4B).

Prostate-specific antigen (PSA, UniProt id P07288, gene name KLK3) was the most significant “hit” with 7.5-fold increase in PCa serum (DDA-TMT data, FDR p.value = 5.4 e-05). PSA was however not detected as significantly changing in DIA-LF plasma samples and it was not possible to calculate p-value and fold change in the DIA-LF serum due to missing values.

Interestingly, pregnancy zone protein (PZP, FC $\sim$ 11, FDR p.value = 2.3e-10) was also upregulated in PCa serum. PZP is predicted to be secreted to blood and is also known to be highly expressed in late-pregnancy serum. Many more proteins were reproducibly identified as downregulated in PCa serum. Notably, IGHM, COLT1 and TAGLN2.

### Comparison of serum vs. plasma profiles and their utility for biomarker discovery

Plasma and serum are both components of blood but differ in their composition due to the presence or absence of clotting factors. We queried the blood coagulation pathway from the PANTHER Pathways dataset (https://maayanlab.cloud/Harmonizome/gene_set/Blood+coagulation/PANTHER+Pathways) and were able to identify 33 out of the 39 participating proteins in both sample types. Interestingly, six of the blood coagulation components (PROC, APP, ITGB3, PROS1, GP1BA, ITGA2B) showed differential expression patterns in serum (control vs. PCa comparisons) while none were detected as changing in plasma. Since serum is formed by allowing blood to clot naturally, leaving behind a fibrin clot, and containing components released during clotting (e.g., clotting factors), it is possible some of the detected changes are due to those processes and could confound detection of biomarker signatures in serum samples.

**Fig. 5:**
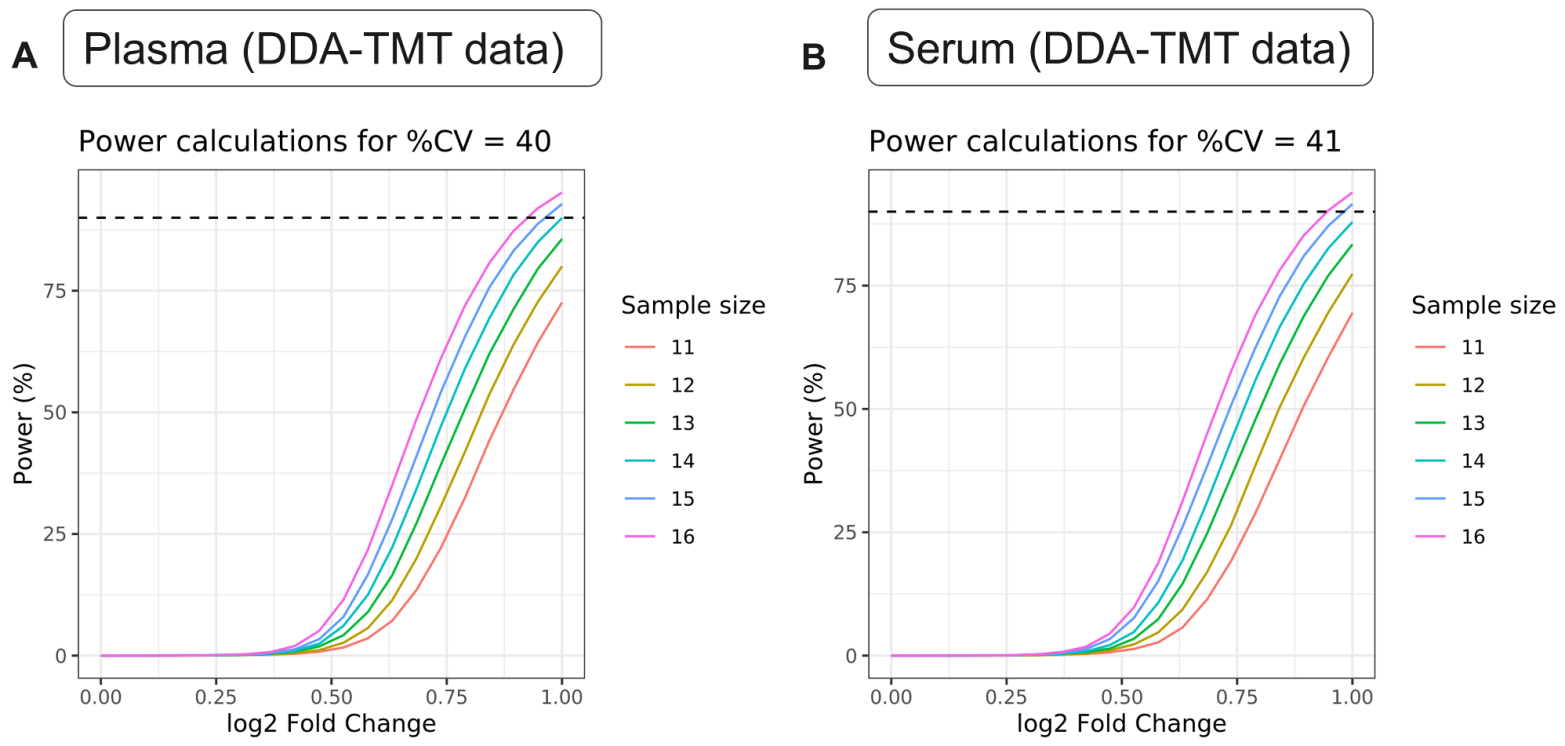
Power curves for detecting a true log2 fold change with varying sample sizes in (**A**) plasma DDA-TMT data and (**B**) serum DDA-TMT data. Each curve represents the statistical power to detect a true difference as a function of log2 fold change for different sample sizes ranging from 11 to 16. The dashed horizontal line represents a 90% power threshold, which is the desired level of power to detect a difference if it truly exists

To assess the feasibility of detecting differential signatures in either biofluid, we performed sample size and power calculations using an in-house omics signatures analysis application. This tool considers the number of features, groups, samples per group, log2 standard deviation of measurements, desired true log2-fold change, and expected number of detectable features at a specified false discovery rate. We achieved very similar power to detect differential expression in both matrices (Fig. 5A and B). This was also consistent for DIA and DDA datasets, albeit DDA data suffered much reduced power overall. For example, using the median standard deviation of all proteins in each matrix, we achieved 90% power (power = 0.9) to detect a fold change of 2 with n = 14 samples per group in plasma DDA-TMT data, and the same fold change required n = 15 in serum DDA-TMT data (Fig. 5A and B). While the differences are small, it appears lower sample sizes are needed to achieve desired statistical power in our plasma samples.

### Prioritising potential markers due to secretion into blood

Numerous proteins are actively secreted by cells in response to diseases or external stimuli, making them valuable candidates for potential biomarkers. Changes in the abundance of such proteins that translocate to the bloodstream can provide real-time insights into the cell’s state and particularly disease progression. To effectively prioritize the numerous differentially expressed proteins we identified in patients with PCa, we set out to determine which of these proteins are actively secreted into the human bloodstream. These are proteins that are deliberately released or secreted into the bloodstream, rather than merely present due to cell death or other disease-related factors.

In pursuit of this, we cross-referenced our list of DEPs with the human secretome catalogue defined by the Human Protein Atlas (HPA) [25, 26].

The Human Protein Atlas is regularly updated, and at the time of our study, it featured predictions for 729 proteins actively translocating to peripheral blood. Notably, we discovered that 57 of the DEPs (quantified with FDR p-values < 0.05 in at least one of the four experiments) are actively secreted into the blood.

Intriguingly, four actively secreted proteins (APOA4, APOB, APOC4, PZP) exhibited significant changes in both serum and plasma of PCa patients and were identified in at least three proteomic datasets. In addition to PSA, FAM3B was the only other differentially regulated protein in our data (log2FC = -1.4, FDR p.value = 0.02 in Serum.DDA.TMT) that was both actively secreted and with RNA specific expression in prostate cancer (40.3 FPKM according to HPA).

## Discussion

With the emergence of new proteomics technologies, including commercial sample preparation kits and state-of-the-art mass spectrometers, the demand for their evaluation and application in specific research contexts becomes increasingly essential. In this study, we conducted a practical assessment of selected aspects, encompassing sample preparation kits, analytical column performance, LC gradient lengths, and the latest data acquisition modes in mass spectrometry. This practical, nonexhaustive, evaluation resulted in the optimization of both low-throughput labeled (DDA-TMT) and higher-throughput label-free (DIA-LF) workflows. Subsequently, we applied these optimized workflows to investigate protein expression changes in the blood of patients with prostate cancer. DIA acquired popularity relatively recently and various DIA acquisition schemes have been developed since then [27–29]. DIA focuses on recording fragments of all detectable peptides present in a sample and enables consistent and accurate protein quantification. Although attractive, a challenge is the intensive computational analysis to deconstruct highly complex fragment spectra that contain several co-eluted and subsequently co-isolated peptides. This approach inherently alleviates the “missing value” problem most prevalent in the stochastic sampling of precursors in DDA approaches. In our case, the single shot DIA workflow resulted in approximately 50% fewer identifications as compared to DDA. This is mainly because of no prefractionation steps and shows that for deep coverage simplifying sample complexity is still preferred. On the other hand, the BoxCar MS acquisition method applied here aims to improve the detection of intact precursor ions. This is accomplished by sequentially filling the quadrupole-orbitrap mass analyzer with different mass windows, which allows increased proteome sampling depth on MS1 level. Typically, the total signal of the orbitrap is concentrated within few peaks representing high analyte concentration which fill the C-trap charge capacity ($\sim$ 1 million charges). Distributing the charge capacity evenly over multiple Thompson (Th) segments across the full mass range limits the accumulation of highly abundant species in the C-trap therefore allowing increased ion injection time for less abundant species.

In our label free DIA acquisition scheme, samples were analysed separately, and protein quantification was based on precursor ion intensities. In contrast, data dependent acquisition using TMT allowed sample multiplexing and quantification was based on the relative intensities of the reporter ions from fragment spectra. TMT quantification accuracy is known to suffer from interference from background ions in the reporter region resulting in ratio compression, i.e., skewing reporter ion intensities towards a 1:1 ratio [30]. Using the SPS MS3 method for data acquisition alleviates most issues with ratio compression and gives more accurate quantification. We found that the correlation of calculated fold changes between DIA and DDA was good, particularly for the proteins with higher statistical confidence. This underscores the importance of applying robust statistical analysis to the data.

To date, proteome changes in PCa blood have been extensively investigated with both MS and non-MS based methods [20, 31, 32]. Many of those studies are reviewed in the following literature. For example, in a genetics-driven study of serum biomarker signatures a shortlist of consistently quantified 39 candidate biomarkers was identified [33]. Those were later prioritised to select best markers. We identified all the shortlisted proteins in our study. A set of 40 regulated proteins were discovered in a panel of patients reflecting PCa progression (PCa null control, benign disease, T1–T2 and T3–T4 stage PCa) [22]. Of those we were only able to detect 11 proteins in our cohort.

We focused our study on blood as its integral role in maintaining homeostasis implies that it reflects an individual’s phenotypic state. Furthermore, the accessibility of blood from patients makes it an attractive source for clinical applications, specifically biomarker discovery, validation and diagnostics. Another easily accessible biofluid which involves non-invasive collection is urine. Urinary proteome has been studied extensively [34] and is a complex mixture with over 6000 protein detected in healthy urine, spanning six orders of magnitude in concentration [35]. Our data acquisition methods could also be applied to measure this attractive biofluid.

Finally, limitations of our study in the context of biomarker discovery include limited sample size and the relatively homogenous cohort. This makes it difficult to assess the future clinical performance of the DEPs we discovered as biomarkers and their diagnostic sensitivity and specificity. Undoubtedly additional studies would be required to (de)-validate our findings.

In conclusion, notwithstanding the limitations, this work serves to showcase recent developments in instrumentation and advancements in the field of proteomics demonstrating increased depth of coverage and throughput for biofluid analysis.

## Materials and methods

### Sample acquisition

Serum and plasma samples, totaling 32 samples, were sourced from subjects with prostate cancer (PCa) and healthy individuals. These were purchased from a commercial vendor. The dataset included 16 serum and 16 plasma samples from PCa patients, matched with samples from healthy volunteers. Demographic and clinical covariates, such as age, Body Mass Index (BMI), Gleason Score, TNM staging, clinical stage, and Prostate-Specific Antigen (PSA) levels, were available. The samples were fully consented for research, adhered to ethical approval with Institutional Review Board (IRB) or Ethics Committee (EC) clearance, and were free from research restrictions. They originated from Russia, were from adult donors in both health and disease states and were fully anonymized with pathogen testing confirming the absence of common pathogens.

### Sample preparation

A total of 70uL of plasma or serum was added directly to the depletion columns Thermo top 14 midi and abundant proteins are depleted using manufacturer’s protocol with modifications as described. The sample was incubated for 2 h on a rotor at room temperature. After depletion the bottom enclosure was twisted off and spun by placing in a 15mL falcon tube at 1000 g for 2 min. The fitrate contained the sample with top 14 proteins depleted in 10mM PBS, 0.02% azide, pH 7.4. This sample is concentrated by buffer exchange using Amicon filters 3 K (Millipore) according to manufacturers protocol.

Proteins are extracted and digested using the Easypep Thermo kit protocol. In brief, 100uL lysis buffer was added to depleted plasma or serum. The samples was reduced and alkylated using 50uL reduction and alkylation solution by incubating the sample at 95^0^C for 10 min. After the sample was cooled to room temperature 50 µL of the reconstituted Trypsin/Lys-C enzyme solution at 0.2ug/uL was incubated with shaking at 37 °C for 1.5 h followed by which another 25uL enzyme was added to complete the digestion. For label free DIA analysis 60uL was used for clean up as descirbed below and the remaining was labeled for TMT analysis.The TMTpro 16 Plex reagent was prepared acording to the manufacturers protocol, 1 mg of label reagent was used per channel. The digestion and TMT reagent were quenched by the addition of 50 µL of 5% hydroxylamine, 20% formic acid solution to each labelling reaction to quench and acidify. Verify pH < 4 using pH paper. This was followed by peptide clean up performed according to the Easy pep manufacturer’s protocol.

Peptide quantification was done according to manufacturers protocol using Pierce peptide quantitation asssay. Each channel for TMT was normalized to pool equal amount of 35ug prior to high pH reversed-phase fractionation for TMT analysis. Fractionation was performed according to manufacturer instruction (https://www.thermofisher.com/order/catalog/product/84868). Each sample was normalized to load equal amounts for label-free DIA analysis.TMT samples were fractionated using a Dionex U3000 using Xbridge Peptide BEH C18 3.5 μm 1 × 250 mm column operating at 0.1mL/min with a 100 min method. For TMT samples 96 fractions were separated and concatenated to 30 based on time, while for the DIA library generation 96 fractions were separated and concatenated to 24 based on time. For the DIA library generation, two separate libraries for normal and cancer was generated.

### Liquid chromatography mass spectrometry (LC-MS)

FAIMS ion mobility and offline- fractionation with real-time search MS3 enabaled TMTpro 16 Plex Quant on the Orbitrap Eclipse:

TMTpro 16 Plex quantitation was carried out using nanoflow reversed phase LC using a Dionex Ultimate 3000 coupled online to an Eclipse Orbitrap MS equipped with a Nanospray Flex Ion Source, integrated with a column oven (PRSO-V1, Sonation, Biberach, Germany) maintained at 50˚C. Spray voltage was set to 2.2 kV, funnel RF level at 40%, and heated capillary temperature at 300˚C. The FAIMS CV was set to -65, intensity threshold of 5e3, precursor fit set to 80% with fit window of 0.7. Isolation window of MS2 was set to 0.7 m/z, HCD activation energy fixed at 32%, rapid scan rate in the ion trap. Real time search included trypsin enzyme, carbamidomethyl on C, TMTpro16plex on K and N-termini with variable modification of oxidation on M. MS3 scans were conducted on 10 SPS precursors using HCD activation energy of 65% and analyzed in orbitrap using 30k resolution using turboTMT.

DIA Acquisition was caried out using nanoflow reversed phase LC using a Dionex Ultimate 3000 coupled online to an Exploris 480 MS equipped with a Nanospray Flex Ion Source, integrated with a column oven (PRSO-V1, Sonation, Biberach, Germany) maintained at 50˚C. Spray voltage was set to 1.6 kV, funnel RF level at 40%, and heated capillary temperature at 300˚C. Peptides were separated using non-linear gradients. Exploris 480 MS method contained three experiments: MS1, tSIM, and tMS2. Briefly, the MS1 scan resolution was set to 120,000 at 200Th with scan range between 350-1650Th and full AGC target at 300% with IT of 50ms. The tSIM experiment contained multiplex ion set to on with 12 maximum number of multiplexed ions. Resolution set to 120,000 at 200Th and AGC target value of 300% with IT of 20ms. Loop count was set to N with *N* = 2. Lastly, for the tMS2 experiment isolation windows, isolation offset was set to off, fixed normalized CE at 27% with resolution set to 30,000 at 200Th. First mass of 250Th, RF lens at 40% and AGC target of 1,000 with IT of 54ms. Spectral libraries were acquired in DDA with MS resolution set to 60,000 at 200Th and full AGC target at 300% with IT of 25 ms. Mass range was set to 350-1650Th. AGC target value for fragment spectra was set to 200% with a resolution of 15,000 and injection times of 22 ms for Top12. Intensity threshold was kept at 2E5 and isolation width set to 1.3Th. Normalized collision energy was set to 27%. Data was acquired in centroid mode using positive polarity.

### Data analysis

TMT data was analysed using Proteome Discoverer using percolator for 1%FDR cut-off against a canonical human UniProt Fasta file with the following settings: enzyme: trypsin up to two missed cleavages, Fixed modifications: Carbamidomethyl (C) TMTpro (N-term, K); variable modifications of: Oxidation (M), acetyl (protein N-term), reporter ion quantifier set to MS3 FTMS.

For DIA data, a spectral library was constructed with Spectronaut against the canonical Human UniProt Fasta file using following settings: fixed modification: carbamidomethyl (C); variable modification: acetyl (protein N-term), oxidation (M); enzyme: trypsin/P with up to two missed cleavages. Mass tolerances were automatically determined by Spectronaut (Biognosys AG, Schlieren, Switzerland) and other settings were set to default. Search results were filtered by a 1% FDR on precursor, peptide, and protein level. Runs were subsequently searched with the library using default settings. Raw mass spectrometry data and results are available in PRIDE repository [36] with identifier PXD046924.

### Electronic supplementary material

Below is the link to the electronic supplementary material.

**Supplementary Material 1:** Sample metadata table

**Supplementary Material 2:** Results of differential expression analysis between PCa and healthy donors

## Data Availability

Associated data and materials not included as supplementary materials in this manuscript can be accessed upon request.
